# Metabolomic Profile and Its Correlation with the Plasmatic Levels of Losartan, EXP3174 and Blood Pressure Control in Hypertensive and Chronic Kidney Disease Patients

**DOI:** 10.3390/ijms24129832

**Published:** 2023-06-07

**Authors:** Ingrid Souza Reis Santos, Manuel Martin-Pastor, Alberto Gomes Tavares Júnior, Kamila Ayres Queiroz, Lílian Grace da Silva Sólon, Francisco Fábio Oliveira de Sousa

**Affiliations:** 1 Graduate Program on Pharmaceutical Sciences, Department of Biological & Health Sciences, Federal University of Amapa, Macapa 68903-419, Brazil; 2 Laboratory of Quality Control, Bromatology and Microbiology, Department of Biological & Health Sciences, Federal University of Amapa, Macapa 68903-419, Brazil; 3 Unidade de Resonancia Magnetica, Área de Infraestruturas de Investigación, Campus Vida, Universidad de Santiago de Compostela, 15072 Santiago de Compostela, Spain

**Keywords:** hypertension, chronic kidney disease, metabolomics, losartan, EXP3174, plasmatic

## Abstract

Systemic arterial hypertension (SAH) is one of the most prevalent chronic diseases worldwide and, when dysregulated, may cause serious complications. Losartan (LOS) blocks relevant physiological aspects of hypertension, acting mainly on the reduction of peripheral vascular resistance. Complications of hypertension include nephropathy, in which diagnosis is based on the observation of functional or structural renal dysfunction. Therefore, blood pressure control is essential to attenuate the progression of chronic kidney disease (CKD). In this study, ^1^H NMR metabolomics were used to differentiate hypertensive and chronic renal patients. Plasmatic levels of LOS and EXP3174, obtained by liquid chromatography coupled with mass-mass spectroscopy, were correlated with blood pressure control, biochemical markers and the metabolomic fingerprint of the groups. Some biomarkers have been correlated with key aspects of hypertension and CKD progression. For instance, higher levels of trigonelline, urea and fumaric acid were found as characteristic markers of kidney failure. In the hypertensive group, the urea levels found could indicate the onset of kidney damage when associated with uncontrolled blood pressure. In this sense, the results point to a new approach to identify CKD in early stages and may contribute to improving pharmacotherapy and reducing morbidity and mortality associated with hypertension and CKD.

## 1. Introduction

SAH is one of the most prevalent chronic diseases worldwide, reaching about 30% of the population. During the early stages, it presents itself silently, while the progression may cause serious complications. Elevated and sustained levels of blood pressure (BP) are indicative for diagnosis, and follow up and continuous treatment are used to control and reduce its complications [1,2].

The antihypertensive drug LOS, an angiotensin-2 receptor antagonist, blocks all relevant physiological mechanisms of hypertension, acting mainly on the reduction of peripheral vascular resistance [3]. Its half-life is increased when metabolized into its active metabolite EXP3174 from 6 to 9 h, which potentiates the blockade of angiotensin II in the vascular smooth muscle, while its peak concentration occurs between 1 and 3 h [4].

LOS can be administered in daily doses of 25–100 mg with good tolerance and few side effects, confirmed by different studies since the1980s [5].

Nephropathy is a cause and major complication of uncontrolled SAH. The diagnosis is based on the observation of functional or structural renal impairment [6]. BP control is essential to attenuate the progression of chronic kidney disease (CKD), as angiotensin mediates the kidney impairment, emphasizing its actions beyond BP control [7].

Some risk factors are related to CKD, such as an age over 50 years, genetic predisposition, male gender, obesity, diabetes and SAH. Some tests are recommended for monitoring kidney damage and are used to determine the stage of CKD, such as a conventional biochemical analysis, urinalysis and renal/urinary tract ultrasound [8]. Nonetheless, in many cases, patients are not aware of the initial disfunction at an early point and late diagnosis often occurs, limiting the recovery and therapeutic options. Kidney dysfunction often causes systemic damages to the organism. For instance, the drugs’ pharmacokinetics may be altered by the inherent disfunction characteristic of the disease, which may be further aggravated by the use of nephrotoxic drugs [9].

Metabolomics come as a novel approach to investigate and propose diseases mechanisms and progression, based on the metabolites’ pathways. This approach allows for analyzing the drug vs. the physiological relationship. More recently, the organism’s responses to external stimuli, obtained from plasma samples and biomarkers, have been used to evaluate the effectiveness of pharmacotherapy [10], pharmacokinetics patterns and nephrotoxicity [11,12], but there are yet few studies bringing this approach to humans to date.

Accordingly, the present study aimed to identify the metabolomic phenotypes of hypertensive and CKD patients and correlate that to the plasmatic levels of LOS and EXP3174 and to the clinical outcomes. This approach not only could serve for a rapid prognostic, even in cases where early clinical signs of nephropathy are not evident, but also for deducing the altered metabolic pathways in the early stages of renal disfunction.

## 2. Results

### 2.1. Participants’ Characteristics

Sixty-seven adult volunteers were recruited for this study. The case-type patients were divided into the following subgroups: SAH—22 hypertensive patients using LOS exclusively and CKD—19 hypertensive and nephropathic patients using LOS exclusively, with 5 renal outpatients (CKA) and 14 undergoing hemodialysis treatment (CKN). Twenty-six control volunteers (CTRL) were also enrolled in the study.

Blood samples were collected from fasting volunteers before and 1.5 h after LOS administration in the SAH group. Patients´ sociodemographic and main clinical characteristics are presented in Table 1.

Patients were adults, majorly women. SAH and CKD patients presented high levels of BP before the administration of LOS, classified as high and very high, respectively. The SAH group was evaluated before and 1.5 h after the administration of LOS, while for the CKD group, the collection was done before the hemodialysis (for those who underwent this treatment—CKN) and the administration of LOS, which occurs just after that. Creatinine and urea were found to be critically above normal values in the CKD patients, while the SAH group also presented values of urea (83.5 mg/dL) much above the reference range. Control patients presented all biochemical parameters within the normal reference range. 

### 2.2. Analytical and Bioanalytical Validation

The method validation for plasma quantification of LOS and EXP3174 was successfully accomplished. The parameters found in the validation of LOS and EXP3174 are displayed in Table S1. Bioanalytical validation (Table S1) confirmed the analytical adequacy. The calibration curves obtained from the bioanalytical phase showed a strong linear relationship, with the R² values greater than 0.99 for both analytes.

The method reached satisfactory precision, with CV values between 0.05 and 4.89% for LOS and EXP3174 in analytical and bioanalytical validation. Accuracy values ranged from 81.33–133.3%. The freeze–thaw cycle used to check the stability of the analytes under thermic stress conditions resulted in recoveries from 127.16% to 127.90% after 8 h and 107.42% to 114.32% after 24 h for LOS and 111.41% to 111.41% after 8 h and from 89.79% to 104.25% after 24 h for EXP3174. Very low detection and quantification limits of LOS (LoD 0.5 ng/mL, LoQ 5 ng/mL) and EXP3174 (LoD 2 ng/mL, LoQ 5 ng/mL) were found (Table S1), confirming the high sensitivity of the method used.

**Figure 1:**
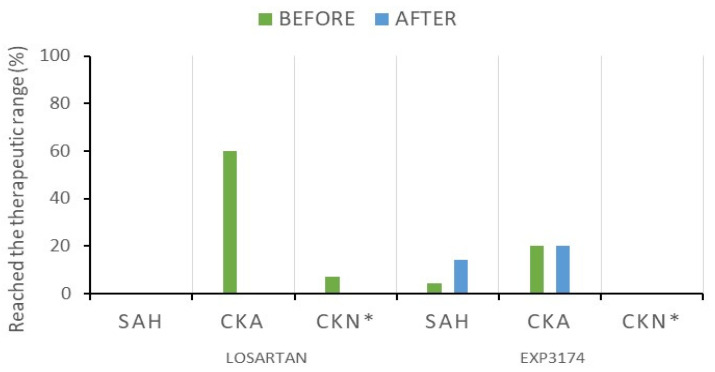
Number of hypertensive and nephropathic patients who reached the therapeutic range of losartan: 200–650 ng/mL and EXP3174: 200–1200 ng/mL; * single blood collection was performed 24 h after drug administration in nephropathic patients undergoing hemodialysis. SAH: Hypertensive patients, CKA: Nephropathic outpatients, CKN: Nephropathic patients undergoing dialytic treatment.

According to the results, the SAH group did not reach therapeutic levels of LOS neither before nor 1.5 h after the oral administration of LOS tablets (Figure 1). The therapeutic levels of EXP3174 were reached by 5 and 14% of the patients before and after administration, respectively. This aspect is not very relevant for EXP3174, as its C_max_ is expected to occur after 3 h upon LOS administration [4].

The plasmatic levels of LOS and EXP3174 found in CKD patients, both outpatients (CKA) and those undergoing dialytic treatment (CKN) (Figure 1), were also outside the therapeutic range of LOS and EXP3174 for most patients, except for a single patient (A_4_) who presented bioaccumulation of LOS, eventually associated with some type of metabolism disorder as it was not converted into EXP3174 (Table S2). The therapeutic level of LOS was reached by 60% in the group CKA and 7% in the group CKN, respectively, indicating that, although both have renal impairment, the second is likely to require doses adjustment. Moreover, the EC_50_ of LOS was reached by 50% of the SAH patients and limited to 21.05% among the CKD patients (Table 1).

### 2.3. NMR Metabolomics

NMR metabolomics, using both a fingerprint and targeting analysis [14,15] of ^1^H_T2, was used. Potential biomarkers related to SAH and CKD were searched. The CTRL group was used as reference. 

In view of that, the results demonstrating significance after satisfying the spectral quality criterion based on PCA, and leading to favorable separation of the groups by a multivariate statistical analysis (MSA), were submitted to an univariate analysis to access first the differences between the three groups and also in pairs. The SAH and CTRL groups were not pair compared, as they did not differ in any buckets, while the group CKD was analyzed also within the two subgroups CKA (outpatients) and CKN (undergoing hemodialysis) to access the differences found in the different stages of renal disease.

**Figure 2:**
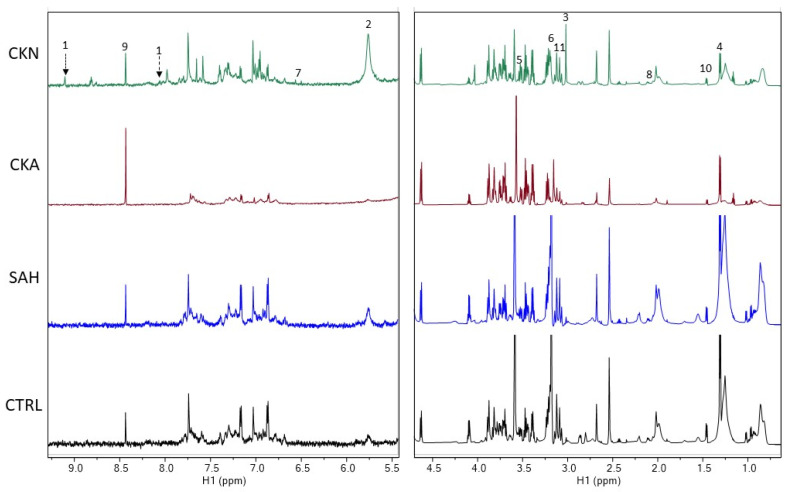
^1^H_T_2_ spectra of plasma samples showing representative metabolites found within the groups. 1 = trigonelline, 2 = urea, 3 = creatinine, 4 = lactate, 5 = glycine, 6 = choline, 7 = fumaric acid, 8 = glutamine, 9 = anserine, 10 = alanine and 11 = proline. CKN: Nephropathic patients undergoing dialytic treatment, CKA: Nephropathic outpatients, SAH: hypertensive patients and CTRL: control group.

Representative spectra of the studied groups with the discriminatory metabolites found in the following analysis are found in Figure 2.

#### 2.3.1. NMR of CTRL vs. SAH vs. CKD Groups

**Figure 3:**
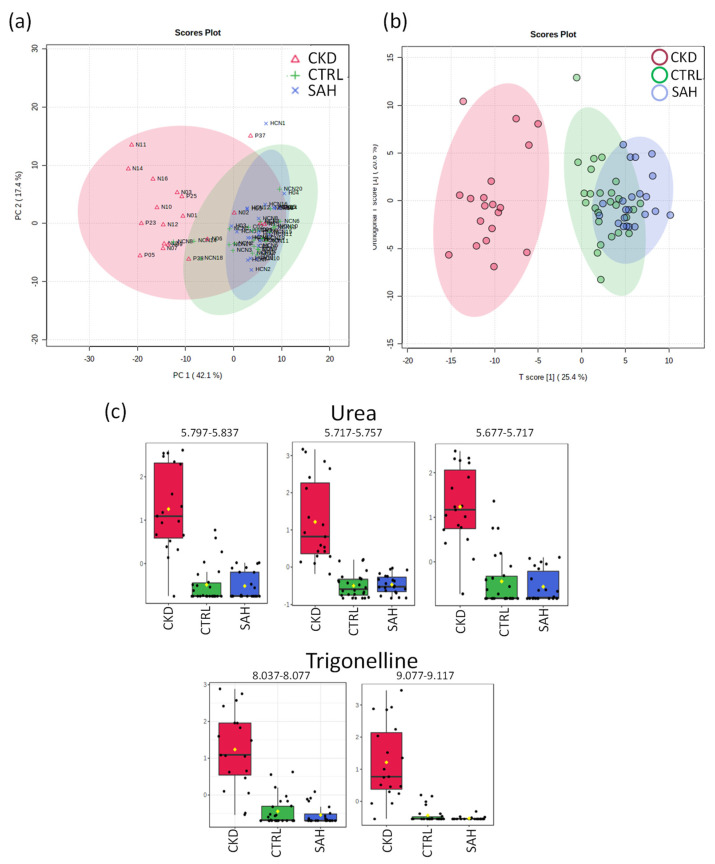
Statistics of targeted analysis of ^1^H_T_2_ spectra of plasma samples from control group (CTRL, *n* = 26) vs. hypertensive patients (SAH, *n* = 22) and hypertensive patients with renal disease (CKD, *n* = 19). (**a**) PCA score plot. (**b**) PLS-DA score plot (R^2^ 0.788 and Q^2^ 0.652, *p* < 5 × 10^−4^). (**c**) Bar graphs of the normalized peak intensities of the identified metabolites with differences between the three groups. Their bucket integrals in the spectra were considered relevant for the PLS-DA classification obtained in (**b**).

The distribution of the PCA score plot for the three groups (Figure 3) is highly compact and relatively random, with no obvious outliers to be considered. The PLS-DA analysis found two regions in the spectrum (buckets) with the maximum discrimination power corresponding to urea and trigonelline (Figure 3c). The score chart in Figure 3b shows the overlapping between SAH and CTRL groups in parallel with a remarkable separation of the CKD group without overlapping. The axes of the 2D scores cover 25.4 and 20.6% of the total variability.

In the analysis of the buckets, two metabolites were identified as the most relevant of PLS-DA: urea and trigonelline. The univariant analysis of the NMR integral in the buckets corresponding to these metabolites in Figure 3c and Table 2 indicates a significant elevation in both metabolites for CKD patients. Trigonelline is an alkaloid product of niacin metabolism, also found in the composition of coffee and teas. Its presence may be related to bioaccumulation after those beverages´ consumption.

#### 2.3.2. NMR of CRTL vs. CKD Groups

**Figure 4:**
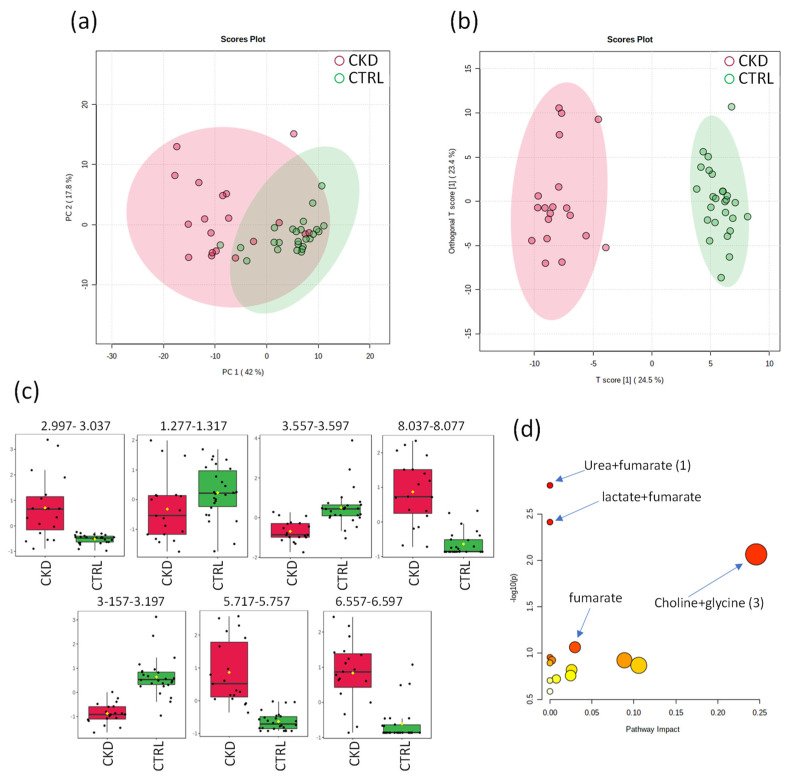
Statistics from targeted analysis of ^1^H_T_2_ spectra of plasma samples from control group (CTRL, *n* = 26) (in green) vs. hypertensive patients with renal disease (CKD, *n* = 19) (in red). (**a**) PCA score plot, (**b**) OPLS-DA score plot (R^2^ 0.964 and Q^2^ 0.861, *p* < 5 × 10^−4^). (**c**) Bar graphs of the distribution of NMR intensities of identified metabolites with differences between the two groups in this order: creatinine, lactate, glycine, trigonelline, choline, urea and fumaric acid. Their bucket integrals in the spectra were considered relevant for the OPLS-DA classification obtained in (**b**). (**d**) Results of the metabolic topology pathway based on potential metabolites in the O-PLS models.

The distribution of the PCA score plot for CTRL and CKD groups (Figure 4) shows considerable differences, emphasizing the peculiarity of CKD. The discriminative metabolites found were creatinine, lactate, glycine, trigonelline, choline, urea and fumaric acid. The score chart in Figure 4b shows the evident separation between CTRL and CKD groups, without overlapping, while the 2D score axes cover 24.5% and 23.4% of the total variability.

A total of seven metabolites were selected as the most relevant biomarkers and their metabolic pathways and hypergeometric testing were analyzed. According to the pathway analysis, the metabolism of urea + fumarate (1) (*p* = 0.0015512; FDR = 0.1303) with the metabolic pathway of arginine biosynthesis; the metabolism of lactate + fumarate (2) (*p* = 0.0038701; FDR = 0.16254) with the metabolic pathway of pyruvate metabolism and the metabolism of choline + glycine (3) (*p* = 0.0086375; FDR = 0.24185) with the metabolic pathway of glycine, serine and threonine metabolism were the three most significant pathways found between the CTRL and CKD groups (Figure 4d).

#### 2.3.3. NMR of SAH vs. CKD Groups

**Figure 5:**
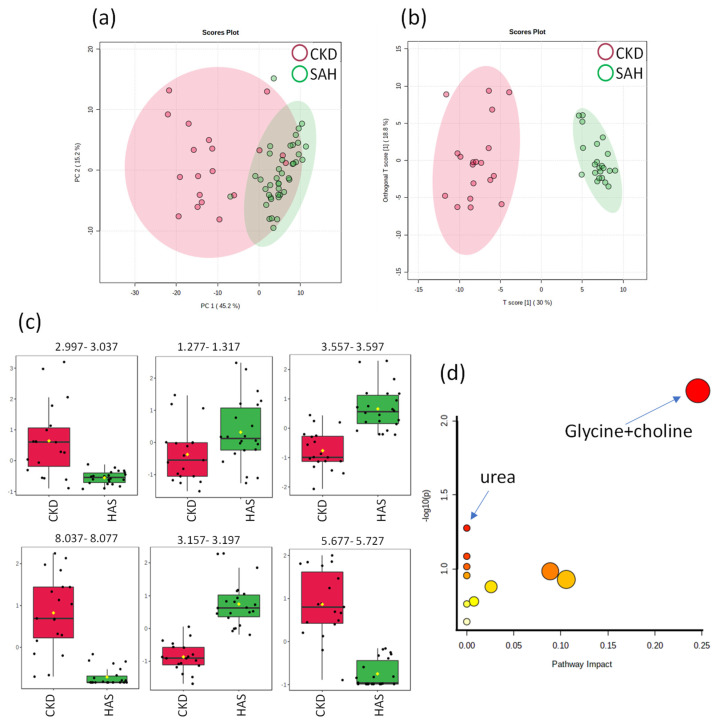
Statistics from targeted analysis of ^1^H_T_2_ spectra of plasma samples from hypertensive patients only (SAH, *n* = 22) (in green) and hypertensive patients with renal disease (CKD, *n* = 19) (in red). (**a**) PCA score plot, (**b**) OPLS-DA score plot (R^2^ 0.950 and Q^2^ 0.863, *p* < 5 × 10^−4^). (**c**) Bar graphs of the distribution of NMR intensities of identified metabolites with differences between the two groups in this order: creatinine, lactate, glycine, trigonelline, choline and urea. Their bucket integrals in the spectra were considered relevant for the OPLS-DA classification obtained in (**b**). (**d**) Results of the metabolic topology pathway analysis based on potential metabolites in the O-PLS models.

The distribution of the PCA score plot for SAH and CKD groups (Figure 5) indicates separation. OPLS-DA found two regions in the spectrum (buckets) with the maximum discriminating power corresponding to the metabolites: creatinine, lactate, glycine, trigonelline, choline and urea. The separation evidenced by the score chart (Figure 5b) demonstrates the clear distinction between hypertensive patients and nephropathic patients. The 2D score axes cover 30 and 18.8% of the total variability.

A total of three metabolites selected as the most relevant biomarkers and their metabolic pathways and hypergeometric testing were analyzed. According to the pathway analysis, the urea metabolism (*p* = 0.053068; FDR = 1.0) with the arginine biosynthesis metabolic pathway and the glycine + choline metabolism (*p* = 0.0062527; FDR = 0.52523) with the glycine, serine and threonine metabolism were the most significant pathways correlated between SAH and CKD groups (Figure 5d).

#### 2.3.4. NMR of CKA vs. CKN Groups

**Figure 6:**
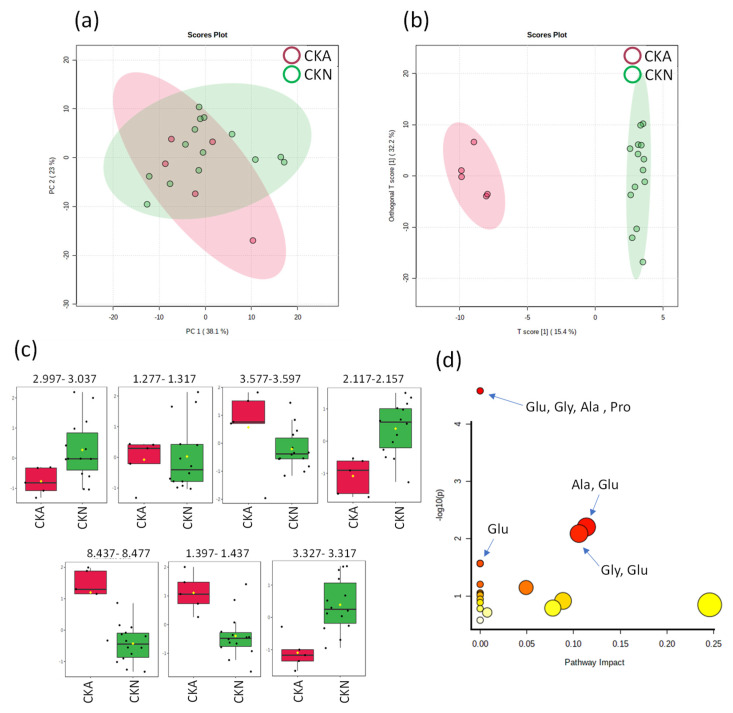
Statistics from the directed analysis of ^1^H_T_2_ spectra of plasma samples from hypertensive patients with renal disease in outpatient follow-up (CKA, *n* = 5) (in red) vs. hypertensive patients with kidney disease undergoing hemodialysis (CKN, *n* = 14) (in green). (**a**) PCA score graph, (**b**) OPLS-DA score graph (R^2^ 0.990 and Q^2^ 0.934, *p* < 5 × 10^−4^). (**c**) Bar graph of the distribution of NMR intensities of the identified metabolites of the two groups in this order: creatinine, lactate, glycine, glutamine, anserine, alanine and proline. Their bucket integrals in the spectra were considered relevant for the OPLS-DA classification obtained in (**b**). (**d**) Results of the metabolic topology pathway analysis based on potential metabolites between CKD subgroups CKA and CKN in the O-PLS models.

The renal patients (CKD) were subdivided according to their disease progression, i.e., CKA are the outpatients and CKN are the patients undergoing hemodialysis treatment. The best MSA classification of the CKA and CKN groups was obtained with the direct analysis of the ^1^H_T_2_ spectra. The distribution of the PCA score plot for the two groups (Figure 6a) is highly compact and relatively random, with no outliers to be considered. The OPLS-DA analysis found seven regions in the spectrum (buckets) with the maximum discrimination power corresponding to creatinine, lactate, glycine, glutamine, anserine, alanine and proline. The score plot in Figure 6b shows an excellent group separation without overlapping and the 2D score axes cover 15.4 and 32.2% of the total variability.

A total of five metabolites selected as the most relevant biomarkers and their metabolic pathways and hypergeometric testing were analyzed. According to the pathway analysis, the metabolism of glutamine + glycine + alanine + proline (*p* = 2.653 × 10^−5^; FDR = 0.0022285) with the Aminoacyl-tRNA biosynthesis metabolic pathway; the metabolism of glycine + glutamine (*p* = 0.0081316; FDR = 0.22768) with the metabolic pathway of metabolism of glyoxylate and dicarboxylate; glutamine metabolism (p1 = 0.026836; FDR1 = 0.45084; p2 = 0.026836; FDR2 = 0.45084) with two metabolic pathways of nitrogen metabolism and D-glutamine and D-glutamate metabolism; and arginine + glutamine metabolism (*p* = 0.006251; FDR = 0.22768) with the metabolic pathway of alanine, aspartate and glutamate metabolism were found as the most significant pathways correlated within the CKD subgroups comparison (Figure 6d).

**Figure 7:**
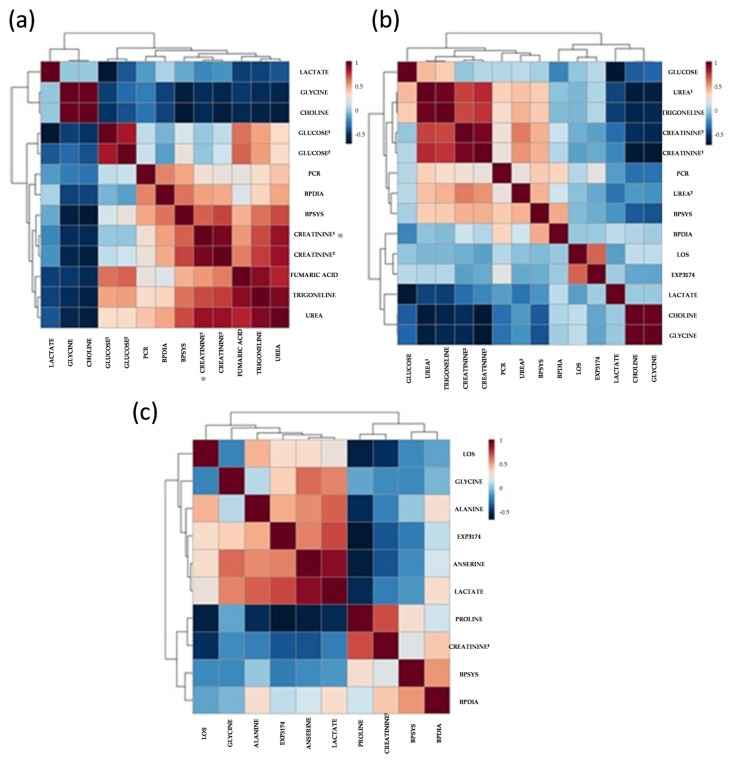
Comprehensive profile of metabolites identified by ^1^H NMR analysis in plasma was analyzed by hierarchical clustering. Clustering method: UPGMA (unweighted average); similarity measure: Euclidean distance; sorting function: average value. The heat map shows the logarithmic ratio of the base 2 to the means of the corresponding compared groups. (**a**) Correlation heat map between the CTRL vs. CKD groups. (**b**) Correlation between SAH vs. CKD groups. (**c**) Correlation between CKA vs. CKN groups. BPSYS—systolic blood pressure, BPDIA—diastolic blood pressure, LOS—plasmatic level of losartan. EXP3174—plasmatic level of EXP3174. ^1^Obtained by ^1^H NMR. ^2^ Obtained by conventional laboratory analysis.

A heat map representation of the correlations made is shown in Figure 7. The hierarchical grouping of metabolite biomarkers quantified in samples for the groups in pairs correlated with the clinical variables, such as BP, plasmatic levels of LOS and EXP3174, and relevant biochemical parameters. Some metabolites, such as creatinine and urea, were found to have high levels in both the biochemical analysis and NMR, validating the results of the metabolomic analysis. Nonetheless, others presented limited levels, such as glycine. A more detailed description of changes observed in the metabolome profile vs. clinical aspects examined will be discussed below.

## 3. Discussion

The determination of biomarkers was performed by ^1^H NMR plasmatic metabolomics. The NMR data were correlated with biochemical parameters, BP control and plasmatic levels of LOS and EXP3174.

The target BP values vary according to the type of population and are also different for the hypertensive and non-hypertensive population. The control group presented BP levels of SBP < 120 and DBP < 80 mm Hg, while the BP of the other two groups (SAH and CKD) showed important changes. The therapeutic target of hypertensive patients is BP ≤ 130/80 mm Hg, except for patients at high cardiovascular risk whose target is reduced to ≤120/80 mm Hg [16].

After EC_50_ is reached, the BP lowering effects are visible. Nonetheless, only 50% of the patients of the SAH group reached a satisfactory value for EC_50_ (Table 1), while a very limited number reached the expected therapeutic range for LOS and EXP3174 (Figure 1), reflected directly in the levels of BP observed. This aspect may be a risk factor for the onset of renal alterations not yet evident/diagnosed among these patients. Therefore, it is important to consider the dose adjustment and monitoring of the plasmatic levels of LOS. In view of the tenuous relationship between hypertension and kidney disease, monitoring should always be considered as an important preventive measure. Early identification of the onset of renal impairment is imperative for conservative measures. Confusing or silent clinical signs are common at the early stages, which leads to a later diagnostic, where the CKD is already installed in a more advanced course [17]. Therefore, clinical laboratory tests are recommended for the diagnosis and monitoring of both SAH and CKD diseases [18].

Regarding the CKD hypertensive patients, the scenario of goals and treatments are modified. The objective is to reduce BP in order to achieve a DBP < 90 mm Hg. Some studies were unable to confirm that lower targets would reduce the risk associated [19].

The CKD group had a mean BP of 164/86 mm Hg (Table 1). In addition, only 21.05% reached the EC_50_ of LOS (Table 1), which is directly reflected in the levels of BP achieved. Uncontrolled BP increases the coronary risk [20]. Therefore, more attention should be given to this group in view of the results found.

In association with these findings, the subtherapeutic levels of LOS and EXP3174 in this group (Table 2) may be associated with the elapsed interval (24 h) between the administration of LOS and the blood collection, which could be within the normal range if the BP values were adequate. Therefore, dosage adjustment or association with other drugs is suggested to adjust the BP level, while the need to monitor LOS in CKD patients is emphasized.

Regarding the biochemical parameters (Table 1), the CTRL group was found to be within the reference values. As for the SAH group, all parameters are within the normal range, except for urea (Table 1). One of the hypotheses for this is the early renal impairment, as SAH is one of the most frequent causes of CKD, accounting for 34% of cases in 2017 [21]. Elevated BP, associated with arterial stiffness, leads to an increase in pressure in the renal irrigation arterioles, interfering with the renal autoregulation process, causing glomerular hyperfiltration and hyperperfusion, slowly deteriorating its functions [22]. Moreover, all biochemical parameters evaluated were found to be altered in the CKD group (Table 1). Remarkably, the high plasmatic levels of creatinine and urea found indicate chronic renal impairment [23] and evidence that despite the dialysis treatment, no significant reduction can be reached in those parameters.

In addition to these findings, the PCR results indicate that none of the groups studied were undergoing an acute infectious phase and/or any inflammatory processes, which could interfere with the other variables studied, especially in the metabolomics patterns.

Based on the comparisons between groups, some metabolites showed good correlation in the PLS-DA analysis. When analyzing the three groups together, two metabolites showed greater relevance, urea and trigonelline, notably increased in the CKD group. Urea is directly related to CKD characteristics. Trigonelline presents itself as an innovative finding, associated with kidney injury, given the high level found in the CKD group. Being a by-product of niacin (vitamin B3) metabolism, trigonelline is, after caffeine, the second largest alkaloid compound found in raw coffee beans, being excreted unaltered in urine [24,25]. Human exposure to trigonelline occurs through diet, consumption of foods such as oats, potatoes and especially coffee; thus, it is also used as a biomarker of coffee consumption [24,26].

Few reports associate this biomarker with injury and/or kidney disease. A study reported trigonelline as a urinary biomarker for renal ischemia and reperfusion in a swine model [27], while another study pointed it as a marker of kidney damage caused by cisplatin in rats [28].

In our investigation, the increase in trigonelline levels in patients with CKD may be associated with its bioaccumulation from the consumption of foods rich in B3, such as coffee, reaffirming previous studies of its association with kidney damage, and therefore, it is a valuable biomarker that could be used in early stages to determine renal impairment in high-risk patients, for instance in diabetic and hypertensive patients.

Urea is synthesized in the liver from ammonia, which comes from amino acids such as arginine. It is the main by-product of protein catabolism and is mostly excreted through the kidney, representing about half of all solid products found in urine [29]. In view of this, urea is another important kidney misfunction marker.

In our study, when evaluating the three groups, urea was also present in higher concentrations in the SAH and CKD groups. Despite not being as specific as creatinine, it is a crucial marker of the initial changes that cause pathologies, because it is more sensitive to primary changes in kidney conditions [30], also confirming its role as a biomarker of kidney injury.

The pathway of arginine and proline metabolism is altered in CKD, as the kidney is directly related to arginine biosynthesis [31]. In our investigation, proline was found at high levels in patients with CKD undergoing hemodialysis (CKN) when compared to the outpatients (CKA), which may be a result of the accumulation of ornithine (an intermediate of the urea cycle) in advanced CKD [32]. Proline also acts as a (weak) antagonist of the glycine receptor and NMDA (N-Methyl D-Aspartate) and non-NMDA [33] ionotropic glutamate receptors. 

Glutamine together with glutamic acid play important roles in ammonia, nitrogen and protein metabolism, being a substrate for gluconeogenesis in the kidney [34,35]. In this study, glutamine was found to be increased in CKN patients. This metabolite is converted into glutamate in the kidney and via gluconeogenesis, into pyruvate. The elevation found in this group may suggest bioaccumulation associated with LOS treatment, which may be indirectly aggravating the CKD due to the chain imbalance that it may cause.

Pyruvate generated by the glutamine chain reaction is further converted to alanine and is a direct participant in muscle energy production [33]. In this study, alanine was found to be reduced in the CKD dialytic group. This can be related to the bioaccumulation of glutamine, impairing the protein biosynthesis in this group. Anserine, also found at low levels in plasma, when broken down is also converted into alanine and similarly may correlate with the low alanine levels observed [33].

The antihypertensive effect of LOS depends not only on its concentration, but also on the metabolites found in the plasma. In this study, the volunteer with the highest plasmatic levels of LOS was not the one with the highest levels of EXP3174 (Table S2), demonstrating how complex the human body is and emphasizing the difficulty in quantifying individual differences based on genetic variations only.

Regarding the metabolomic analysis, no significant differences in the biomarkers among the CTRL and SAH groups were found. In contrast, remarkable differences were found between the CKD group when correlated with the two other groups. Lactate was reduced in patients with CKD compared to CTRL and SAH. In the study of He et al. [11], lactate demonstrated a positive correlation with the Cmax ratio achieved after LOS administration in non-hypertensive patients [11]. Higher levels of plasma lactate as well as a hyperglycemic response were also found in rats in which LOS was administered intravenously, suggesting that the angiotensin AT1 receptor may alter the energetic balance [36].

Creatinine was elevated in CKD patients compared to the other groups. Additionally, in the study of He et al. [11], this metabolite was found to have a negative correlation with AUC. It is assumed that the pharmacokinetic response of LOS, notoriously impaired in our findings, could be in part related or linked to plasmatic levels of creatinine, which bioaccumulates due to the inherent characteristic of renal disease. Moreover, high serum creatinine levels would be related to poor survival and a lower response to specific treatments in kidney disease [37,38]. This metabolite has already been characterized as a marker of target organ damage and a predictor of cardiovascular risk in hypertensive patients [39]. The higher its value, the lower the conversion of LOS into EXP3174 [40]. Therefore, creatinine and lactate showed strong correlations with characteristics of LOS metabolism and kidney disease, corroborating the findings of He et al. [11].

Comparing the levels of these two metabolites within the subgroups of patients with CKD (CKA—outpatients and CKN—undergoing dialysis) was also relevant. It is known that kidney damage caused by acute kidney injury can result in CKD. Elevated levels of creatinine, urea, sodium and lactate at emergency admission are independent risk factors for death in patients with acute kidney injury [41], corroborating our findings, as a direct relationship was found between the degree of renal injury and the plasma levels of these metabolites.

In the study of He et al. [11], the metabolism of glycine, serine and threonine was significantly associated with the AUC/Cmax ratio, with glycine and choline being the most negatively involved metabolites. In another study, an animal model of hypertension showed that the regulation of glycine, serine and threonine metabolism occurs from the antihypertensive effects of the drug [42]. When evaluating cardiovascular risk, choline was found to be related to the increased risk of cardiovascular diseases [43], while glycine, which is a constituent of collagen and its antioxidant effects, has been inversely associated with hypertension [44]. When comparing these findings to our data, higher levels of choline and glycine were found in the plasma of SAH compared to the CKD group. Therefore, it could increase the cardiovascular risk of this group and might be affecting directly the metabolism of LOS. However, when comparing renal outpatients (CKA) with those undergoing dialysis (CKN), glycine levels were higher in the first group, which may suggest that the permanence of this variable may lead to the progression of the disease as it affects the metabolism of LOS.

Another relevant metabolite found was fumaric acid, which showed higher levels in patients with CKD compared to the CTRL group. This metabolite requires conjugation with glycine as a pathway for its excretion [33]. As glycine was found to be reduced in these patients, it may have contributed to the increased plasmatic level of this metabolite. Therefore, fumaric acid could also be an indirect biomarker of kidney disease.

In view of the results presented, it can be noted that NMR metabolomics allowed for the finding of relevant biomarkers for significant differentiation among CTRL, SAH and CKD groups, despite the reduced number of volunteers in the study. The analysis of these biomarkers can clarify different mechanisms, being useful to observe the correlation of biomarkers with the prediction, alterations and stage of SAH and CKD. Nevertheless, it is important to emphasize that the proposal is made with groups in which some variables are not under control (real patients) and that the pathophysiology can vary, for instance in the group with CKD undergoing dialysis treatment (CKN). Population-based studies are suggested to confirm those findings and to validate the aspects presented herein.

## 4. Materials and Methods

### 4.1. Study Design

An observational, case-controlled study, with a quantitative approach and spontaneous sampling of sixty-seven adult patients, was carried out. After the informed consent form was signed, blood samples were obtained from hypertensive (SAH) (*n* = 22)/CKD-hypertensive (*n* = 19) patients. Healthy volunteers (*n* = 26) were also enrolled as a control group (CTRL) for comparison. BP measurements were done immediately before the blood collections. A semi-structured questionnaire was used to inquiry the volunteers about their social, clinical profile (age, sex, other diseases and BP).

The SAH group was evaluated before (fasted) and 1.5 h after the administration of LOS, while the CKD group was evaluated before the administration of LOS and before dialysis for those who were undergoing this treatment. This group was not evaluated after administration, due to the expected plasmatic drug loosening due to the dialysis and the difference between the administration regimens according to medical prescription. The CTRL group only had a single collection (fasted). The BP levels were measured just before the blood collections. The hypertensive participants underwent supervised oral administration of LOS—50 or 100 mg (according to their individual medical prescription) with 100 mL of water. The CTRL group only received the same volume of water. Fresh blood samples were collected in a 5 mL heparinized tube. After 30 min at room temperature, the samples were centrifuged at 3000 rpm for 10 min and the plasma was aliquoted into plastic cryogenic microtubes and stored at −40 °C for further biochemical analysis (urea, creatinine and C-reactive protein—PCR) and plasma quantification of LOS and EXP3174. The study was approved by the Research Ethics Committee of the Federal University of Amapa (CEP/UNIFAP) under CAAE nº 18337719.8.0000.0003.

### 4.2. Sample Preparation for UHPLC-ToF-MS Plasmatic Quantification

A previous method validated by our group was used to determine the plasmatic levels of LOS and EXP3174 [45], according to ICH Q2 (R1) guidelines [46], including analytical and bioanalytical validation. The parameters’ linearity, precision (repeatability), accuracy, limit of quantification and detection, robustness, selectivity and recovery were determined for the analytical stage [45], while linearity, precision (repeatability), accuracy, limits of quantification/detection and ice-thaw cycles in enriched human plasma were used to validate the proposed method for plasmatic quantification of LOS and EXP3174.

Analytical standards of LOS, losartan acid (EXP3174) and the internal standard (IS, irbesartan) (Synfine^®^, Richmond Hill, ON, Canada) were used.

The samples were analyzed in an Ultra High Performance Liquid Chromatograph (Agilent^®^, Santa Clara, CA, USA), coupled to a TOF mass spectrometer (Bruker^®^, Billerica, MA, USA), with chemical ionization at atmospheric pressure (APCI) and a time-of-flight analyzer (TOF) in positive mode.

Sample preparation before the analysis followed protein precipitation protocols. The plasma obtained from the patients was spiked with the internal standard irbesartan. The analytes were extracted from 300 μL of plasma, which was vigorously mixed with 340 µL of acetonitrile (HPLC grade) and centrifugated at 3000 rpm for 10 min at 4 °C. The supernatant was filtered in a PTFE syringe filter (0.22 µm) and injected into the equipment. 

### 4.3. Sample Preparation and Acquisition of ^1^H NMR Spectra

The samples for the metabolomics analysis were prepared according to the method used by Beckonert et al. and Dona et al. [14,47]. An aliquot of 300 μL of plasma and 300 μL of a bibasic sodium phosphate heptahydrate buffer (Na_2_HPO_4_·7H_2_O) were homogenized and centrifuged at 10,000 rpm for 10 min. The supernatant was used to obtain the NMR spectra on a Bruker NEO, 17.6 T spectrometer (proton resonance 750 MHz), equipped with a ^1^H-^19^F/^13^C/^15^N triple resonance PA-TXI probe with a deuterium lock channel and shielded PFG z-gradient. The spectrometer control software was TopSpin 4.0. The chemical shifts reported are referenced to the lock deuterium solvent. Spectra were processed and analyzed with Mestrenova^®^ software v14.0 (Mestrelab Inc., Escondido, CA, USA).

Carr-Purcell-Meiboom-Gill (CPMG) spin-echo sequence experiments (^1^H-T_2_ spectra) were performed with 128 scans, a total spin–spin relaxation time of 2.5 s, spectrum size of 32 K, acquisition time of 1.95, a spectral width of 8389.26 Hz, 90° for the rotation angle of the radiofrequency pulse and a line amplification factor of 0.3 Hz. All spectra were acquired at 27 °C using a sequence solvent pre-saturation pulse, to suppress the water signal, and referenced with trimethylsilyl propanoic acid-TSP.

The ^1^H-T_2_ spectra were aligned, baseline corrected, normalized based on the total spectral area and divided into 81 bands of equal width [(Δδ) 0.04 ppm], between 0.8 and 8.5 ppm using Mestre Nova^®^ software version 12.0. The spectral region comprised between 4.7 and 5.2 ppm was removed due to variability in the suppression of the water signal. A representative spectrum of the classification was loaded in ChenoMX NMR Suite software v8.5 (Chenomx^®^, Inc., Edmonton, AB, Canada) and confirmed by the human metabolome database (HMDB) if found in human plasma and with a concentration ≥ 20µM, and categorized in the metabolomine if common or uncommon in the MRI bank plasma metabolism (BMRB) and related literature. A receptor operating characteristic (ROC) analysis was performed to assess the diagnostic accuracy of each candidate biomarker and a panel of predictive biomarkers that showed the highest accuracy was presented based on the combinational ROC analysis. A heat map methodology was also used to correlate the metabolites to the analytical and relevant clinical aspects.

### 4.4. Metabolic Pathway Analysis

To assess the biological roles of the identified metabolites and identify overrepresented pathways in the metabolite list, we used the web-based Metaboanalyst 3.0 (https://www.metaboanalyst.ca/, accessed on 1 December 2022) and the Metabolic Biological Role (MBRole) (https://csbg.cnb.csic.es/mbrole2/, accessed on 1 December 2022). These are online platforms for metabolite annotation enrichment analysis that calculate the *p*-values with the cumulative hypergeometric distribution by comparing the number of compounds in the set and in the background with a given annotation [48]. Values of *p* < 0.05 were considered significant. The Human Metabolome Database, Biological Magnetic Resonance and Bruker Biofluid Reference Compound Database Library and the Metaboanalyst 4.0 software were used to confirm the identity, the metabolomic pattern and its correlation with the pharmaco-toxicological parameters of LOS and its EXP3174.

Although the studies of He et al. [11] and Uehara at al [12] do not explore a pathway, the remarkable metabolites were also used as a reference for correlating with the pharmacokinetic and clinical aspects: LOS and EXP3174 plasmatic levels, BP values and eventually any sign of renal injury, given the limited number of studies addressing these issues. 

### 4.5. Statistic Analysis

The data obtained from the questionnaires were tabulated using Microsoft^®^ Excel 2010 for Windows and submitted to a descriptive analysis. The numerical values obtained from the metabolomic analysis were processed by the orthogonal signal correction technique (OSC) [49] and also analyzed with the Metaboanalyst 3.0 [50,51]. The O-PLS model was applied to correlate NMR data with the biomarkers’ identification. 

Two types of multivariate methods were used for the NMR bucket data. The first method is the unsupervised principal component analysis (PCA) which was used for the detection of anomalous or outlier samples that have to be discarded because they do not satisfy the quality criteria, possibly due to errors during sample collection, preparation, storage and/or NMR measurement. The NMR data of the samples not discarded by PCA were subsequently analyzed by the MSA method of the Orthogonal-Partial Least Squares-Discriminant Analysis (OPLS-DA). Based on their pattern of intensities, those spectral buckets that best discriminate between groups provided the highest values of VIP scores and loading factors in OPLS-DA. A random test with 2000 permutations was performed with the derived OPLS-DA model to confirm the robustness of the method for a model based on the two main components describing the variability of the samples, resulting in the reported values of R^2^ and Q^2^. R^2^ = 1 indicates perfect description of the data by the model and Q^2^ = 1 indicates perfect predictability. In general, Q^2^ > 0.5 and the difference between R^2^ and Q^2^ < 0.3 demonstrate good predictability [52]. Cross-validation was applied to the OPLS-DA model obtained to determine its accuracy and to derive the confusion matrix of the classification achieved.

A univariate analysis was carried out to study the signal intensity of selected NMR buckets with the highest VIP scores and loading factors found in the MSA calculation by OPLS-DA. They are the buckets with the maximum relevance for the group classification achieved by the MSA method. The selected buckets were those with a loading factor comprised of a range from the maximum down to 50% of this value. The distribution of NMR intensities of each of the selected buckets were represented as Boxplots and the *p* value of the distribution was calculated with the unpaired *t*-test. The normality of the data was tested by the Kolmogorov–Smirnov normality test. If data underwent the Gaussian distribution performed with Welch’s correction, it was assumed that the standard deviation was not equal, or otherwise, the Mann–Whitney test was used.

Metabolite assignment was carried out for each NMR bucket that satisfied the same criterion of selection of the univariate analysis, to identify plausible candidates as biomarkers of the disease. The experimental signal profile and intensity of the chemical shift of the bucket were then matched to the ^1^H patterns of the 750 MHz metabolite ChenoMX^®^ NMR Suite software v8.5 database. A metabolite candidate is obtained when the bucket chemical shift, intensity and signal profile (i.e., pattern of J couplings) can be matched to a given signal of a metabolite in the spectral database, and simultaneously, other additional ^1^H peaks of the metabolite match are compatible with the experimental spectrum. Only those metabolites with favorable matching, and that could be assigned unambiguously, were reported.

## 5. Conclusions

The NMR metabolomics study of plasma samples from hypertensive and hypertensive chronic kidney disease volunteers identified several biomarkers that distinguished the groups of patients. Remarkably, higher levels of trigonelline, urea and fumaric acid compared to the other groups were considered characteristic markers of renal impairment. For patients with systemic arterial hypertension, urea levels were also likely to mark the onset of kidney injury when associated with the high values of blood pressure found.

Although the number of subjects was limited within the groups, the statistical model predicted with adequate robustness. 

Hypertensive and renal patients presented high blood pressure levels before LOS administration, and were classified as high and very high, respectively. Additionally, the EC_50_ of LOS was achieved by solely 50% of hypertensive patients and limited to 21.05% among renal patients, while the therapeutic range was reached by a very limited number of patients in both groups, except for the renal outpatients.

Regarding the biochemical tests, creatinine and urea were found critically above the normal range among the renal patients, while the hypertensive patients also presented urea values well above the reference range.

The differences found by the NMR metabolomic analysis among the groups were in agreement with the blood pressure values and provided relevant and consistent data on the pharmacometabolomic profile and the distinction between the hypertensive and chronic kidney disease groups. The latter was also differentiated according to the different progression levels of the disease. Clinical protocols for the therapeutic monitoring of LOS can be created from the proposed methodology, efficiently assisting in pharmacological treatment, especially for high-risk patients, such as hypertensive and diabetic patients, to avoid or limit the renal impairment in early stages. Moreover, renal patients can improve the treatment outcomes, avoiding the progressive dysfunction caused by the disease, especially in outpatients, while increasing the quality of life and reducing the expenses related to this clinical condition.

Personalized medicine to improve the therapeutic strategies and individual follow-up is a modern and necessary approach. In view of the various possibilities that this type of study represents and the results found, there is a need for further investigation, including populational-based studies covering this topic and complementing the findings to understand the mechanisms involved in hypertension, kidney disease, their relationship and to also validate the results found using a new cohort. This improvement would make this methodology useful for clinical practice to assess the response based on molecular biomarkers.

## Figures and Tables

**Table 1:** Demographic and main clinical characteristics of the study participants (mean ± SD).

Variable	CTRL (*n* = 26)	SAH (*n* = 22)	CKD (*n* = 19)
Age (years)	43.9 ± 10.5 ^a^	54.5 ± 11.6 ^a^	54.2 ± 11.2 ^a^
Female gender (%)	61.54	45.45	50.75
Others diseases (%)	0.0	9.09	52.63
BPSYS (mmHg) ^1^	116 ± 9.6 ^b^	B:143 ± 18.0 * A:138 ± 18.7 ^b^	164 ± 27.0 *
BPDIA (mmHg) ^1^	75 ± 8.5 ^c^	B:91 ± 11.0 ^c,^* A:83 ± 11.7 ^c^	86 ± 18.8 ^c^
Patients with plasmatic levels > EC50 (%) ^a^	-	50.0	21.05
Creatinine (mg/dL) ^2^	0.8 ± 0.2 ^d^	0.9 ± 0.2 ^d^	10.2 ± 5.2 *
Urea (mg/dL) ^2^	23.8 ± 5.5	83.5 ± 30.8 ^e,^*	109.1 ± 34.2 ^e,^*
PCR (mg/dL) ^2^	1.8 ± 3.4 ^f^	2.0 ± 3.1 ^f^	3.6 ± 3.9 ^f^

CTRL = control group, SAH = hypertensive group, CKD = nephropathic group. ^a^ efficiency concentration (EC_50_) of losartan is 32 µg/L [13]. B: before and A: after. ^1^ Reference for normal blood pressure values: Systolic blood pressure (BPSYS) ≤ 120 mm Hg and Diastolic blood pressure (BPDIA) ≤ 80 mm Hg. ^2^ Complementary tests for biochemical comparison between studied groups. Biochemical markers reference values for adults. Creatinine: 0.40–1.30 mg/dL; Urea: 15–45 mg/dL; C—reactive protein: ≤6.0 mg/L. * The average is above the normal values. Data were submitted in pairs to one-way ANOVA with a post hoc Tukey test when *p* < 0.05. Same letters in each line indicates no statiscical significante between the groups (*p* > 0.05).

**Table 2:** Relevant metabolites identified by NMR metabolomics in CTRL vs. SAH vs. CKD on plasma samples.

METABOLITE (NMR Bucket, ppm)	HMDB ID	CKD	SAH	CTRL
Urea	HMDB0000294			
(5.79–5.83)		High	Intermediary	Low
(5.71–5.75)		High	Low	Intermediary
(5.67–5.71)		High	Intermediary	Low
Trigonelline	HMDB0000875			
(8.03–8.07)		High	Low	Intermediary
(9.07–9.11)		High	Low	Intermediary

## Data Availability

Not applicable.

## Supplementary Materials

The following supporting information can be downloaded at: https://www.mdpi.com/article/10.3390/ijms24129832/s1.
