# Supplementary material for: Metabolomic Profile and Its Correlation with the Plasmatic Levels of Losartan, EXP3174 and Blood Pressure Control in Hypertensive and Chronic Kidney Disease Patients
Source: Int J Mol Sci. 2023 Jun 7;24(12):9832. doi: 10.3390/ijms24129832 (PMC10298398; doi:10.3390/ijms24129832)
Supplement: Supplementary file 1 [file ijms-24-09832-s001.zip › ijms-2135199-supplementary.pdf]

Table S1. UHPLC-ToF-MS Losartan and EXP3174 determination in acetonitrile HPLC grade (analytical method) and human plasma matrix (bioanalytical method) validation.

**Losartan - Validation parameters ng/mL**		**Analytical validation**	**Bioanalytical validation**		
**Linearity (n = 3)**		R^2: 1.00000		R^2: 0.99689	
		y = 2*107x –9021.5		y = 0.5862x + 2.641	
**Sensitivity**		LoQ: 5		LoQ: 5	
		LoD: 2		LoD: 0.5	
		% ± SD		% ± SD	
**Accuracy (n = 9)**	[1,510]	92.8 ± 0.05	[750]	81.33 ± 0.20	
	[750]	102.69 ± 0.03	[150]	86.67 ± 0.05	
	[25]	99.4 ± 0.0007	[30]	133.3 ± 0.06	
		Mean ± %CV		Mean ± %CV	
**Precision (n= 9)**	[1,510]	1.39 ± 4.22	[750]	0.61 ± 0.20	
	[750]	0.770 ± 4.89	[150]	0.13 ± 0.05	
	[25]	0.025 ± 3.08	[30]	0.17 ± 1.33	
		%Change			
**Robustness (n = 3)**	[750][a]	-5.26			
	[750][b]	-14.57			
				% after 8h	% after 24h
**Ice-thaw cicle**			[750]	127.16	107.42
			[30]	127.90	114.32

EXP3174 - Validation parameters ng/mL		Analytical validation		Bioanalytical validation	
**Linearity (n = 3)**		R^2: 0.9966		R^2: 0.99843	
		$y = 1*10^7x – 257159$		$y = 0.1825x + 0.09865$	
**Sensitivity**		LoQ: 10		LoQ: 5	
		LoD: 4		LoD: 2	
		% ± SD		% ± SD	
**Accuracy (n = 9)**	[2,775]	91.58 ± 0.04	[925]	82.16 ± 0.23	
	[925]	89.42 ± 0.02	[185]	91.89± 0.08	
	[278]	88.85 ± 0.001	[37]	108.11 ± 0.10	
		Mean ± %CV		Mean ± %CV	
	[2,775]	2.541 ± 1.59	[925]	0.76 ± 0.23	
**Precision (n= 9)**	[925]	0.827 ± 3.29	[185]	0.17 ± 0.08	
	[278]	0.247 ± 0.50	[37]	0.04 ± 0.10	
		%Change			
**Robustness (n = 3)**	[925[a]]	-6,80			
	[925[b]]	-10,8			
				% after 8h	% after 24h
**Ice-thaw cicle**			[925]	124.21	89.79
			[37]	111.41	104.25

[a]*Acetronitrile HPLC supra gradient grade,* [b]*non-agitated.*

Table S2. Plasmatic levels of Losartan and EXP3174 of hypertensive and CKD volunteers.

PATIENT SAMPLE	LOSARTAN (ng/mL)		EXP3174 (ng/mL)	
SAH	Before administration	After administration	Before administration	After administration
H_{1}	-	56	8	72
H_{2}	-	88	8	46
H_{3}	-	12	30	22
H_{4}	-	64	48	88
H_{5}	-	76	14	24
H_{6}	-	48	-	44
H_{7}	-	72	-	22
H_{8}	-	40	-	12
H_{9}	-	54	8	40
H_{10}	58	-	112	22
H_{11}	-	80	-	14
H_{12}	-	82	-	228[1]
H_{13}	-	-	-	-
H_{14}	-	100	26	118
H_{15}	-	72	-	14
H_{16}	-	152	14	48
H_{17}	-	164	8	12
H_{18}	-	62	2	10
H_{19}	-	-	-	-
H_{20}	10	178	210[1]	444[1]
H_{21}	-	-	-	-
H_{22}	74	70	156	336[1]

CKD	Before administration	After administration	Before administration	After administration
OUT (CKA)				
A_{1}	290[1]	4	469.6[1]	18
A_{2}	2	4	198	242[1]
A_{3}	296[1]	6	182	26
A_{4}	582[1]	26	18	14
A_{5}	38	-	26	24
DIA[2] (CKN)				
N_{1}	-		-	
N_{2}	-		-	
N_{3}	-		16	

N_4	-	26
N_5	252[1]	38
N_6	-	-
N_7	-	-
N_8	-	48
N_9	-	56
N_{10}	-	6
N_{11}	30	150
N_{12}	-	-
N_{13}	-	-
N_{14}	18	192

Results were calculated using the calibration curve and expressed as mean (in ng/mL). Therapeutic range of losartan: 200 – 650ng/mL and EXP3174: 200 – 1200ng/mL; OUT – outpatients; DIA – undergoing hemodyalisis; - Not detected; [1]reached the therapeutic range; [2]a single blood collection was performed 24h after drug administration in nephropathic patients undergoing hemodialysis. H_1-H_{22}: Hyperthensive patients, A_1-A_5: Nephropatic outpatients, N_1-N_{14}: Nephropatic patients undergoing dyalitic treatment.
